# Human ESCs predisposition to karyotypic instability: Is a matter of culture adaptation or differential vulnerability among hESC lines due to inherent properties?

**DOI:** 10.1186/1476-4598-7-76

**Published:** 2008-10-03

**Authors:** Puri Catalina, Rosa Montes, Gertru Ligero, Laura Sanchez, Teresa de la Cueva, Clara Bueno, Paola E Leone, Pablo Menendez

**Affiliations:** 1 Andalusian Stem Cell Bank/University of Granada, Instituto de Investigación Biomédica, Granada, Spain

## Abstract

**Background:**

The use of human embryonic stem cells (hESCs) in research is increasing and hESCs hold the promise for many biological, clinical and toxicological studies. Human ESCs are expected to be chromosomally stable since karyotypic changes represent a pitfall for potential future applications. Recently, several studies have analysed the genomic stability of several hESC lines maintained after prolonged *in vitro *culture but controversial data has been reported. Here, we prompted to compare the chromosomal stability of three hESC lines maintained in the same laboratory using identical culture conditions and passaging methods.

**Results:**

Molecular cytogenetic analyses performed in three different hESC lines maintained in parallel in identical culture conditions revealed significant differences among them in regard to their chromosomal integrity. In feeders, the HS181, SHEF-1 and SHEF-3 hESC lines were chromosomally stable up to 185 passages using either mechanical or enzymatic dissection methods. Despite the three hESC lines were maintained under identical conditions, each hESC line behaved differently upon being transferred to a feeder-free culture system. The two younger hESC lines, HS181 (71 passages) and SHEF-3 (51 passages) became chromosomally unstable shortly after being cultured in feeder-free conditions. The HS181 line gained a chromosome 12 by passage 17 and a marker by passage 21, characterized as a gain of chromosome 20 by SKY. Importantly, the mosaicism for trisomy 12 gradually increased up to 89% by passage 30, suggesting that this karyotypic abnormality provides a selective advantage. Similarly, the SHEF-3 line also acquired a trisomy of chromosome 14 as early as passage 10. However, this karyotypic aberration did not confer selective advantage to the genetically abnormal cells within the bulk culture and the level of mosaicism for the trisomy 14 remained overtime between 15%–36%. Strikingly, however, a much older hESC line, SHEF-1, which was maintained for 185 passages in feeders did not undergo any numerical or structural chromosomal change after 30 passages in feeder-free culture and over 215 passages in total.

**Conclusion:**

These results support the concept that feeder-free conditions may partially contribute to hESC chromosomal changes but also confirm the hypothesis that regardless of the culture conditions, culture duration or splitting methods, some hESC lines are inherently more prone than others to karyotypic instability.

## Background

Human embryonic stem cells (hESCs) hold the promise not only in cell replacement therapies but also in basic research in a variety of fields ranging from developmental biology, drug screening and toxicology, nutritional reprogramming and disease modelling [1,2]. Human ESCs are derived from the inner cell mass of surplus embryos [1,2]. Once established, hESC lines are desired to be chromosomally stable. The existence of chromosomal abnormalities in hESCs is an important concern, given that karyotypic changes are often associated with carcinogenesis and impaired *in vitro *and *in vivo *cellular behaviour, compromising hESC-based downstream applications. Thus, for the potential of hESC-based therapy to be realized, it is essential that these valuable cells be proven safe and stable.

Over the last 3–4 years controversial data has been reported regarding the chromosomal integrity of hESCs maintained after prolonged *in vitro *culture [3-11]. Some laboratories [3-8] independently showed chromosome changes in H1, H7, H14, HS181, HS237, SA002.5, hESC5 and BG01 hESC lines; the changes emerged in most cases beyond passage 13. In contrast, other studies [4,9-11] reported a lack of karyotypic changes in a variety of hESC lines (SA001, hES1-6, BG02, BG03, SA003, SA121, SA461, HS235) grown between 34 and 140 passages. This cytogenetic resilience of some hESC lines [4,9-11] clearly differs from previous studies [3-8]. These controversial findings are believed to stem from particular aspects of cell culture methods: i) passage methods; ii) presence versus absence of feeders and iii) duration of the long-term culture.

Regarding the passage method, it has been hypothesized that mechanical passage of hESCs by cutting the colonies into small pieces may contribute to the perpetuation of the euploid population, reducing the appearance of aneuploid clones which seem more common upon enzymatic or chemical passage methodology [9,10]. The mechanism/rationale behind the idea that mechanical cutting of the hESC colonies with subsequent destruction of many single cells within the colony is less stressful and detrimental for the hESC culture than enzymatic-based passage methods widely used with multiple stable primary stem cell subsets including hematopoietic stem cells, mesenchymal stem cells or neural progenitors among others, still needs to be proven and elucidated. Similarly, the culture adaptation of hESCs maintained over a feeder layer upon being transferred to a feeder-free culture system has been proposed as an alternative variable which may, to some extent, favour the appearance of chromosomal changes. As for the duration of the long-term culture, it has also been proposed that older (that is, later passage) cells are more susceptible to karyotypic changes than earlier passage cultures. The question of how many passages hESCs may be expanded without undergoing chromosomal abnormalities seems, however, somewhat academic; in some hESC lines (i.e hESC5) karyotypic insults have been shown to occur at relatively very early passages. Therefore, the relevance and actual biological effects of different culture conditions and the duration of the culture on the chromosomal stability of hESCs still remains to be elucidated.

It must be taken into account that *in vitro *culture of hESCs is an abnormal condition. *In vivo*, the cells of the late inner cell mass to which hESCs correspond to, do not persist but disappear as embryogenesis progresses. Importantly, hESCs are derived from the inner cell mass of surplus embryos which commonly harbour chromosomal abnormalities [12-14]. Recent studies [13,14] reported high rates (about 50%) of aneuploidy observed in human blastocysts originating from preimplantation surplus embryos. Among these abnormal human embryos, most are mosaic (mixture of diploid and aneuploid cells). Blastocysts donated for establishment of hESC lines are commonly derived either from fresh preimplantation embryos of suboptimal quality which are not used for *in utero *transfer or, more commonly from frozen embryos.

We hypothesize that, the existence of over 50% of aneuploid human blastocysts, along with the emergence of karyotypic changes in only about 20% of the hESC lines available and grown in a number of different experienced laboratories suggests that some hESC lines may be more prone to karyotypic instability than others.

In the present study, we have prospectively compared using molecular cytogenetic tools the chromosomal integrity of three hESC lines, HS181, SHEF-1 and SHEF-3, maintained in the same laboratory using identical culture conditions and passage methods. Although feeder-free culture conditions may partially contribute to hESC chromosomal changes, we provide evidence supporting the proof-of-principle that regardless of the culture conditions, culture duration or passage methods, some hESCs are inherently more vulnerable than others to karyotypic instability.

## Results

**Figure 1:**
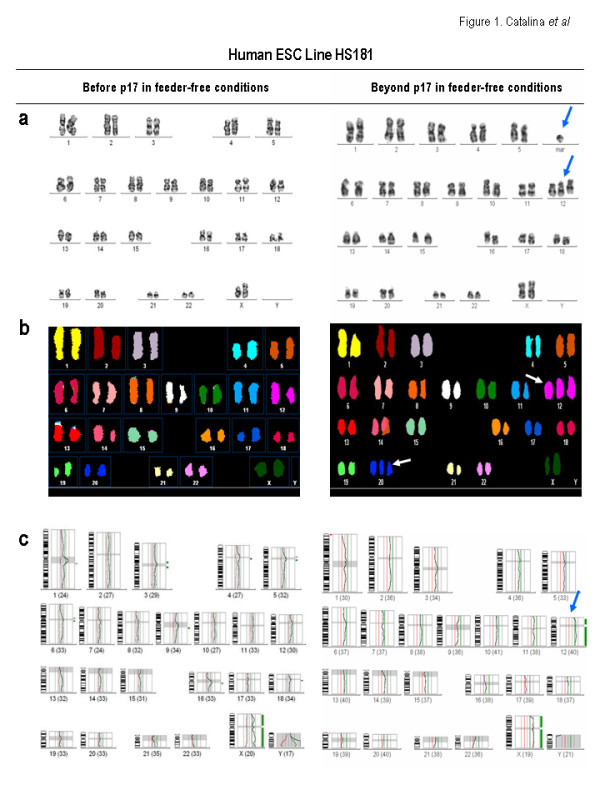
**Molecular cytogenetic analysis of HS181 hESC line**. HS181 hESC line was genetically stable for 71 passages in feeders. Upon being transferred to feeder-free conditions and split using enzymatic methods, the HS181 hESC line gained a chromosome 12 as early as passage 17 and an additional marker, shown by SKY to be an extra chromosome 20 by passage 21. The level of mosaicism gradually increased from 26% (p17) to 89% (p30). In conventional karyotyping analysis (A) and CGH (C), the blue arrows indicate chromosomal gains. In the SKY images (B), the white arrows confirm the gain of chromosome 12 and characterized the marker seen by G-banding as a partial gain of chromosome 20.

**Figure 2:**
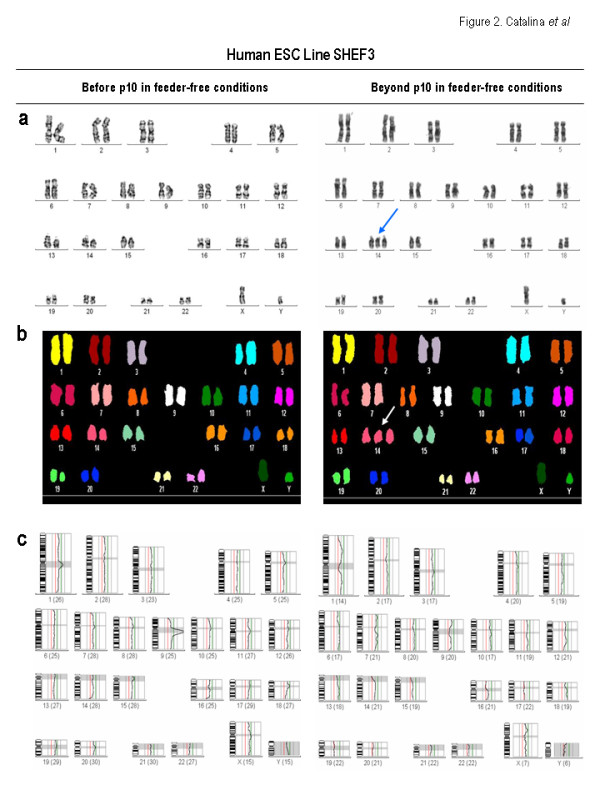
**Molecular cytogenetic analysis of SHEF-3 hESC line**. SHEF-3 hESC line was genetically stable for 51 passages in feeders. Upon being transferred to feeder-free conditions and passaged using enzymatic methods, it quickly gained a trisomy of chromosome 14 as early as in p10. Opposite to the HS181 line, the degree of mosaicism for the extra chromosome 14 did not increase overtime in feeder-free conditions and remained between 23%–36% over the culture (see Table 1). In conventional karyotyping analysis (A), the blue arrows indicate chromosomal gains. B,C: SKY and CGH analyses showing the absence of further structural or numerical abnormalities.

In the present study, we show by means of molecular cytogenetics (G-Banding, Spectral Karyotyping [SKY] and Comparative Genomic Hybridization [CGH]) that both, HS181 and SHEF-3 hESC lines [5,6,15] maintained over a layer of irradiated human foreskin fibroblasts (HFFs) feeder cells and split using mechanical methods proved to be karyotypically stable upon 71 and 51 passages, respectively (Figure 1 &2 and Table 1). Both hESC lines were euploid and neither structural chromosome changes nor gains/losses of DNA content were observed (Figure 1 &2 and Table 1). These cells were then transferred to a feeder-free culture (matrigel-coated surface and HFF-conditioned media [HFF-CM]) and were enzymatically split by using Collagenase IV. The chromosome integrity was determined at different culture time points (p10, p17, p21 and p30). In a feeder-free culture system, the HS181 cells were cytogenetically normal up to p10. Afterwards, they gained an extra chromosome 12 as early as p17 in 26% of the cells comprising the culture. By p21 a marker was detected by conventional karyotyping, characterized as a partial gain of chromosome 20 by SKY (Figure 1 & Table 1). Moreover, the trisomy 12 was found in 31% of the cells by p21. The mosaicism for trisomy 12 gradually increased and by p30, 89% of the cells within the HS181 hESC culture were 47XX + 12, although the extra chromosome 20 eventually disappeared (Figure 1 and Table 1). The chromosomal abnormalities seem to be in line with the ones previously reported in other hESC lines, affecting chromosomes 12 and, at a lesser extent chromosome 20. The cytogenetic analysis carried out at different culture passages (p10, p17, p21 and p30) provided evidence that key genes in specific chromosomes (i.e. chromosome 12) may promote stem cell self-renewal at the expense of differentiation, providing a selective proliferative/survival advantage as it is shown by the outgrowth of the karyotypically abnormal HS181 hESCs over their karyotypically stable counterparts, eventually taking over the hESC culture. In contrast, other karyotypic changes in distinct chromosomes (i.e. an extra chromosome 20 observed in HS181 hESCs) may result in a delayed cell cycle and hence a slower proliferation rate, with those abnormal cells within the hESC cultures eventually disappearing. Similarly, the SHEF-3 line also acquired a trisomy of chromosome 14 as early as passage 10. However, this karyotypic aberration does not seem to confer selective advantage to the genetically abnormal cells within the bulk culture and the level of mosaicism for the trisomy 14 remained over time between 15%–36%. Importantly, these chromosomally unstable hESC lines retained the canonical undifferentiated hESC phenotype (SSEA3+, SSEA4+, Tra-1-60+, Tra-1-81+, Oct3/4+, Nanog+, Rex-1+, Sox-2+), and the ability to differentiate into tissues representing the three germ layers in vivo by teratoma formation (data not shown).

**Figure 3:**
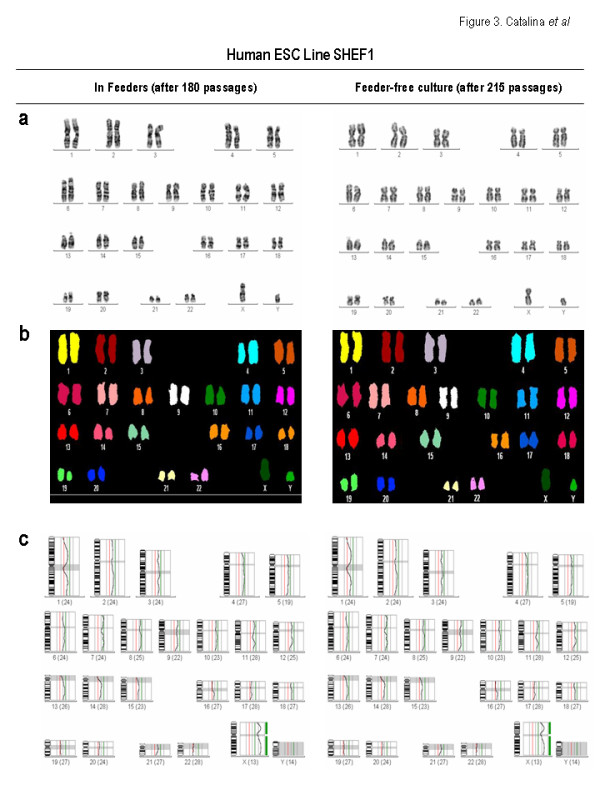
**Molecular cytogenetic analysis of SHEF-1 hESC line**. In contrast to HS181 and SHEF-3 hESC lines, the SHEF-1 hESC line was genetically stable after as many as 185 passages in feeders. Upon being transferred to feeder-free conditions, the SHEF-1 hESC line displayed no karyotypic changes assessed by G-banding (A), SKY (B) and CGH (C) after further 30 passages in feeder-free conditions despite being split by means of enzymatic methods throughout over 215 passages (almost 4 years in *in vitro *culture). This data suggests that although culture conditions may partially contribute to chromosome stability, some hESC lines are inherently more predisposed than others to karyotypic changes.

Despite the fact that these data may throw more light on the genetic stability of hESCs and its relation to how cells are maintained in culture, caution is required when arguing that culture conditions/duration may promote chromosomal aberrations. For instance, Inzunza *et al *[8], Buzzard *et al *[9] and Caisander *et al *[11] showed karyotypic changes in 4 out of 13 hESC lines maintained on mouse or human feeders by mechanical dissociation. Moreover, Draper *et al *[3] reported karyotypic changes in hESCs maintained with or without MEFs and split either mechanically or enzimatically [3]. Imreh *et al *[5] also reported the gain of trisomy 12 in HS181 hESC line maintained in HFFs but passaged using enzymatic methods. Interestingly, we show that in contrast to the HS181 and SHEF-3 hESC lines, a much older hESC line, SHEF-1, which had been maintained for 185 passages in feeders (130 passages in MEFs and 55 passages in HFFs) displayed no karyotypic changes as determined by G-banding, SKY and CGH even after 30 passages in feeder-free conditions despite being split by means of enzymatic methods throughout a total of 215 passages (almost during 4 years) (Figure 3 and Table 1). This data indicates that although culture conditions might partially contribute to chromosomal instability, some hESC lines are inherently more predisposed than others to karyotypic changes, being susceptible to karyotypic abnormalities regardless the presence/absence of feeders, splitting techniques and the duration of the culture. Therefore, this suggests that some hESCs are inherently more prone than others to genomic instability.

## Discussion

Human ESCs have been hailed as a unique tool for biomedical applications such as cell replacement therapy, developmental biology, drug discovery and disease modeling [2,20]. They have the potential to become a powerful tool for modeling different aspects of cancer biology that cannot otherwise be addressed by patient sample analyses or animal models [2,20]. Many hESC downstream applications, however, warrant their culture under feeder-free conditions while retaining pluripotency and genomic integrity; otherwise, cooperating mutations already present could prime hESCs susceptible to cellular transformation [2,20]. Our observations of chromosomal changes occurring only in specific hESC lines not only suggest caution when designing novel culture conditions, and especially feeder-free conditions but should encourage hESC researchers to perform regular high-resolution molecular and cytogenetic studies to verify the chromosome integrity in hESCs using not only G-banding but also CGH, SKY and more precise techniques such as SNP assays to be able to detect tiny but biologically relevant single nucleotide polymorphisms.

Over the last years controversial data has been reported regarding the chromosomal integrity of hESCs maintained after prolonged *in vitro *culture [3-11]. Some laboratories [3-8] independently showed chromosome changes in several hESC lines whereas other studies [4,9-11] reported a lack of karyotypic changes in a variety of hESC lines. These controversial findings are believed to stem from particular aspects of cell culture methods: i) mechanical versus enzymatic passage methods; ii) presence versus absence of feeders and iii) duration of the long-term culture. In the present study, we have compared side-by-side three different hESC lines maintained in identical culture conditions and found significant differences among them in regards to their chromosomal integrity. All three hESC lines were chromosomally stable when grown in feeders. However, despite being maintained under identical conditions, each hESC line behaved differently upon being transferred to a feeder-free culture system. The two younger hESC lines, became chromosomally unstable shortly after being cultured on feeder-free conditions whereas a much older hESC line did not undergo any numerical or structural chromosomal change after >215 passages. While these results are in line with the idea that feeder-free conditions may partially contribute to hESC chromosomal changes they also confirm the hypothesis that regardless of the culture conditions, culture duration or splitting methods, some hESC lines are inherently more prone than others to karyotypic instability. Importantly, the fact that two relatively "young" hESC lines became karyotypically abnormal by passage 61–87 coupled to the genomic stability retained by a much older (over 215 passages) hESC line is clear evidence that the duration of the *in vitro *culture does not seem to contribute to hESC genomic instability.

These data support the notion that chromosomal aberrations seem to occur when hESCs are transferred to and maintained in a feeder-free culture system and that this phenomenon seems to vary among different hESC lines, suggesting that hESC predisposition to karyotypic instability depends on differential vulnerability of distinct hESC lines due to inherent properties rather than just a cell culture adaptation process. Much work is still irequired in order to identify the scope of the problem underlying the karyotypic instability and to unravel the intrinsic and/or extrinsic features which contribute to making some hESCs more prone to chromosomal instability than others. Systematic inter-laboratory comparisons about to what extent inherent intrinsic properties among multiple hESC lines, cytogenetic makeup of the human pre-implantational embryos and hESC derivation methods may contribute to the propensity to karyotypic changes are required. We should bear in mind that *in vitro *culture of hESCs is an abnormal condition. *In vivo*, the cells of the late inner cell mass to which hESCs correspond to, do not persist but disappear as embryogenesis progresses. However, hESCs are derived from the inner cell mass of surplus embryos which commonly harbour chromosomal abnormalities [12-14]. Recent studies [13,14] reported 50% rates of aneuploidy observed in human blastocysts originating from preimplantation surplus embryos. Among these abnormal human embryos, most are mosaic (mixture of diploid and aneuploid cells). Blastocysts donated for establishment of hESC lines are commonly derived either from fresh preimplantation embryos of suboptimal quality which are not used for *in utero *transfer or, more commonly from frozen embryos. This poses many unresolved questions about hESC *in vitro *culture and genomic stability: i) Are hESCs derived from frozen embryos more prone to genomic instability than those derived from fresh embryos? ii) If 40%–50% of the human IVF embryos are karyotypically abnormal, why almost 100% of the newly derived hESC lines are euploid? Is this because karyotypically abnormal embryos cannot progress *in vitro *into a hESC line? iii) Are only the euploid blastomeres in IVF mosaic embryos responsible for colony outgrowth and hESC establishment? iv) Are hESC lines originally derived in a feeder-free system more vulnerable to chromosomal changes than those derived on feeders?

Recent elegant work indicates that cancer may arise from tissue stem cells in adults, and that many cell-signalling pathways essential for normal development (e.g. BMP, Notch, Wnt and Hedgehog) are involved in cancer progression, suggesting a link between embryonic cells and cancer cells [2,22]. The fact that cellular transformation manifests as a blockage or altered cell differentiation suggests that in vitro hESC differentiation could become a promising tool for studying cancer biology and the emergence of transformation events by characterizing the genetic and epigenetic mechanisms that drive cell transformation rather than normal cell specification. Accordingly, it is worth mentioning that the derivation and/or establishment of hESCs carrying specific chromosome abnormalities or characterized mutations may represent an unprecedented tool to dissecting cellular and molecular mechanisms underlying cancer biology by likely disrupting the balance between self-renewal, differentiation and cell death. In fact, our data reveal how distinct cytogenetic abnormalities result in different biological effect. For instance, extra copies of key genes in specific chromosomes (i.e. chromosome 12) may promote hESC self-renewal at the expense of differentiation, providing a selective proliferative/survival advantage as shown by the outgrowth of the karyotypically abnormal hESCs over their karyotypically stable counterparts, eventually taking over the hESC culture. In contrast, extra copies of genes in other chromosomes (i.e. chromosome 14 or 20) might result in either no effect or a delayed cell cycle and hence a slower proliferation rate, with those abnormal cells within the hESC cultures eventually disappearing.

## Methods

### Human ESC culture

The human ESC lines HS181, SHEF-1 and SHEF-3 were maintained either in tissue-treated T25 flasks (BD Biosciences, Bedford, MA) over a confluent (0.5–1 × 10^5 ^feeder cells/cm^2^) layer of X-ray inactivated (4000 rads) HFFs (ATCC; SCD-1112SK) or MEFs in hESC media consisting of 80% KO-DMEM supplemented with 20% KO Serum replacement, 1% nonessential amino acids, 1 mM L-Glutamine, 0.1 mM β-mercaptoethanol and 8 ng/mL of basic fibroblast growth factor (bFGF) (all from Invitrogen, CA) [16] or cultured in Matrigel (BD Biosciences)-coated T25 flasks in human foreskin fibroblast (HFF)-conditioned medium (HFF-CM) supplemented with 8 ng/mL bFGF [17]. To maintain undifferentiated growth, the media (hESC media or HFF-CM) was changed daily, and the cells were split (1:2) weekly by dissociation with 200 U/mL of collagenase IV (Invitrogen).

### Conventional karyotyping

hESCs were cultured in medium supplemented with 0,1 mg/mL colcemid (Biological Industries) for up to 3–4 hours. The cells were then washed in Versene solution (Gibco) and subsequently trypsinized and spun down. The pellet was resuspended carefully in a KCL hypotonic solution (0,075 mol/L), rinsed to remove the cytoplasm, and then fixed in methanol/acetic acid 3:1. The fixing procedure was repeated three times. Finally, the pellet was resuspended in a final volume of 1 mL of fixative, and the cells were dropped onto glass slides. Chromosomes were visualized by using a modified Wright's staining. At least 25 metaphases were analyzed for each cell line using a conventional microscope and the IKAROS-software (Metasystems) [18,19].

### Comparative genomic hybridization

Genomic DNA from hESCs was obtained using the Qiagen DNA isolation kit. Human ESC DNA labeling with fluorescent (Spectrum Green) dUTP was performed using Nick Translation according to Vysis's protocol. Human male gDNA labeled with Spectrum Red was used as reference. After labeling, the length of the DNA fragments were verified by gel electrophoresis to ensure they ranged from 300–3000 bp.

Human ESC labeled-DNA and the reference labeled-DNA were simultaneously hybridized to normal metaphase chromosomes in the presence of Cot-1 DNA to block repetitive sequences. After CGH hybridization in a moist chamber for 48 h, the slides were washed once in 0,4 × SSC/0,3% NP-40 at 73°C for 2 minutes, followed by 2 × SSC/0,1% NP-40 at RT for 1 minute. The slides were air-dried in the darkness and the chromosomes counterstained with DAPI. Twenty metaphases were captured and evaluated for each cell sample [18]. Red/Green ratios higher than 1.25 indicated deleted regions and ratios below 0.80 indicated amplified regions. The CGH quality control test was analyzed using a 99.5% confidence interval. For data analysis, an automated fluorescence microscope system (Nikon Eclipse 50i) equipped with appropriate filters and Metasystem CGH software program was used [8,18,19]

### Spectral karyotyping

For SKY analysis, slides were hybridized using the SKY method according to the manufacturer's protocol (Applied Spectral Imaging, Migdal Ha'Emek, Israel). Images were acquired with an SD300 Spectra Cube (Applied Spectral Imaging) mounted on a Zeiss Axioplan microscope using a custom-designed optical filter (SKY-1; Chroma Technology, Brattleboro, VT). Twenty metaphases were analyzed for each sample. SKY is somewhat limited in the determination of breakpoints and in the identification of intrachromosomal changes such as duplications, deletions, and inversions [8,18-21]. As a result, breakpoints on the SKY-painted chromosomes were determined by comparison of corresponding DAPI banding and by comparison with G-banding karyotype of the hESC line.

## List of abbreviations

hESC: Human Embryonic Stem Cell; CGH: Comparative Genomic Hybridization; SKY: Spectral Karyotyping; SNP: Single Nucleotide Polymorphism; HFF: Human Foreskin Fibroblast; bFGF: basic Fibroblast Growth Factor.

## Competing interests

The authors declare that they have no competing interests.

## Authors' contributions

PC designed and performed experiments and analyzed the data. RM, GL, LS, TdlC and CB performed experiments and analyzed data. PEL provided critical insights, assist in experiments and partially supported the study. PM conceived and supported the study, designed the experiments, analyzed the data and wrote the paper. All authors revised and gave feedback to the last draft.

## Supplementary Material

Additional file 1 Click here for file

## References

[B1] Thomson JA, Itskovitz-Eldor J, Shapiro SS, Waknitz MA, Swiergiel JJ, Marshall VS, Jones JM (1998). Science.

[B2] Bueno C, Montes R, García-Castro J, Greaves M, Menendez P (2008). Drug Discovery Today: Disease Models.

[B3] Draper JS, Smith K, Gokhale P, Moore HD, Maltby E, Johnson J, Meisner L, Zwaka TP, Thomson JA, Andrews PW (2004). Nat Biotech.

[B4] Maitra A, Arking DE, Shivapurkar N, Ikeda M, Stastny V, Kassauei K, Sui G, Cutler DJ, Liu Y, Brimble SN, Noaksson K, Hyllner J, Schulz TC, Zeng X, Freed WJ, Crook J, Abraham S, Colman A, Sartipy P, Matsui S, Carpenter M, Gazdar AF, Rao M, Chakravarti A (2005). Nat Genetics.

[B5] Imreh MP, Gertow K, Cedervall J, Unger C, Holmberg K, Szöke K, Csöregh L, Fried G, Dilber S, Blennow E, Ahrlund-Richter L (2006). J Cell Biochem.

[B6] Gertow K, Cedervall J, Unger C, Szöke K, Blennow E, Imreh MP, Ahrlund-Richter L (2007). J Cell Biochem.

[B7] Pera MF (2004). Nat Biotech.

[B8] Inzunza J, Sahlen S, Holmberg K, Strömberg AM, Teerijoki H, Blennow E, Hovatta O, Malmgren H (2004). Mol Hum Reprod.

[B9] Buzzard JJ, Gough NM, Crook JM, Colman A (2004). Nat Biotech.

[B10] Mitalipova M, Rao RR, Hoyer DM, Johnson JA, Meisner LF, Jones KL, Dalton S, Stice SL (2005). Nat Biotech.

[B11] Caisander G, Park H, Frej K, Lindqvist J, Bergh C, Lundin K, Hanson C (2006). Chromosome Res.

[B12] Hardarson T, Caisander G, Sjogren A, Hanson C, Hamberger L, Lundin K (2003). Hum Reprod.

[B13] Hanson C, Caisander G (2005). APMIS.

[B14] Wilton L (2002). Prenat Diagn.

[B15] Inzunza J, Gertow K, Stromberg AM, Matilainen E, Blennow E, Skottman H, Wolbank S, Ahrlund-Richter L, Hovatta O (2005). Stem Cells.

[B16] Hovatta O, Mikkola M, Gertow K, Strömberg AM, Inzunza J, Hreinsson J, Rozell B, Blennow E, Andäng M, Ahrlund-Richter L (2003). A culture system using human foreskin fibroblasts as feeder cells allows production of human embryonic stem cells. Hum Reprod.

[B17] Menendez P, Wang L, Chadwick K, Li L, Bhatia M (2004). Retroviral transduction of hematopoietic cells differentiated from human embryonic stem cell-derived CD45(neg)PFV hemogenic precursors. Mol Ther.

[B18] Catalina P, Cobo F, Cortes J, Nieto A, Cabrera C, Montes R, Concha A, Menendez P (2007). Conventional and molecular cytogenetic diagnostic methods in stem cell research: a concise review. Cell Biol Int.

[B19] Menendez P, Wang L, Bhatia M (2005). Genetic manipulation of human embryonic stem cells: a system to study early human development and potential therapeutic applications. Curr Gene Ther.

[B20] Catalina P, Montes R, Nieto A, Ligero G, Sanchez L, Jara M, Rasillo A, Orfao A, Cigudosa J, Hovatta A, Greaves M, Menendez P (2008). Scientific and clinical opportunity for modeling infant/childhood leukemia using genetically stable human embryonic stem cells. Leuk Res.

[B21] Gale RE, Hills R, Kottaridis P, Srirangan S, Wheatley K, Burnett AK, Linch DC (2005). No evidence that FLT3 status should be considered as an indicator for transplantation in acute myeloid leukemia (AML): an analysis of 1135 patients, excluding acute promyelocytic leukemia, from the UK MRC AML10 and 12 trials. Blood.

[B22] Menendez P, Bueno C, Wang L (2006). Human embryonic stem cells: A journey beyond cell replacement therapies. Cytotherapy.

