# Supplementary material for: Human ESCs predisposition to karyotypic instability: Is a matter of culture adaptation or differential vulnerability among hESC lines due to inherent properties?
Source: Mol Cancer. 2008 Oct 3;7:76. doi: 10.1186/1476-4598-7-76 (PMC2567976; doi:10.1186/1476-4598-7-76)
Supplement: Additional file 1 [file 1476-4598-7-76-S1.doc]

**Table 1: Summary of the karyotypic changes observed overtime in the HS181, SHEF-1 and SHEF-3 hESC lines and its potential relation to the culture conditions.**

| Human  ESC Line | Passages  under **feeders** conditions | Passages  under **feeder-free** conditions | Karyotype  (G-Banding) | % Mosaicism | **SKY / CGH** |
| --- | --- | --- | --- | --- | --- |
| **HS181** | 71* | 0 | 46,XX | n.a | normal/normal |
|  | 71* | 10 | 46,XX | n.a | normal/normal |
|  | 71* | 17 | 47,XX, +12 | 25 % | n.d |
|  | 71* | 21 | 48,XX, +12, +mar | 31% | 48,XX, +12, +mar |
|  | 71* | 30 | 47,XX, +12 | 89 % | 47,XX, +12 |
| **SHEF-3** | 29** + 22* | 0 | 46,XY | n.a | Normal/normal |
|  | 29** + 22* | 10 | 47,XY+14 | 36% | 47,XY+14 |
|  | 29** + 22* | 17 | 47,XY+14 | 24% | n.d |
|  | 29** + 22* | 21 | 47,XY+14 | 23% | n.d |
|  | 29** + 22* | 26 | 47,XY+14 | 13% | 47,XY+14 |
| **SHEF-1** | 130** + 55* | 0 | 46,XY | n.a | Normal/normal |
|  | 130** + 55* | 10 | 46,XY | n.a | Normal/normal |
|  | 130** + 55* | 17 | 46,XY | n.a | n.d |
|  | 130** + 55* | 21 | 46,XY | n.a | n.d |
|  | 130** + 55* | 30 | 46,XY | n.a | normal/normal |

*Feeders were Human Embryonic Fibroblast (HFFs). **Feeders were Mouse Embryonic Fibroblasts (MEFs).

Abbreviations: n.a: not applicable; n.d: not done; SKY: Spectral Karyotyping; CGH: Comparative Genomic Hybridization
